# The role of whole lung lavage in pneumoconiosis patients with pulmonary diffusion limitation

**DOI:** 10.3389/fmed.2026.1901675

**Published:** 2026-08-12

**Authors:** Qiang-Zhong Pi, Fu Xiong, Lan Liang, Pan Liu, Liang Chen, Bao-Ping Li, Ling Mao, Xiao-Tian Dai, Hai-Qin Guo

**Affiliations:** 1 Department of Respiratory and Critical Care Medicine, The First Affiliated Hospital of Army Medical University (Southwest Hospital), Chongqing, China; 2 Respiratory Department, Beijing Jingmei Group General Hospital, Beijing, China; 3 Occupational Disease Department, Coal General Hospital (Emergency General Hospital), Beijing, China; 4 Occupational Disease Department, Tongji University Affiliated Shanghai Pulmonary Hospital, Shanghai, China

**Keywords:** DLCO, occupational lung disease, pneumoconiosis, pulmonary diffusion capacity, whole-lung lavage

## Abstract

**Background:**

Pneumoconiosis, an occupational lung disease caused by the inhalation of mineral dust, leads to progressive pulmonary fibrosis and impaired gas exchange. A reduced diffusing capacity for carbon monoxide (DLCO) serves as an independent prognostic marker. Although whole lung lavage (WLL) is employed clinically to remove retained dust, its efficacy in ameliorating diffusion dysfunction remains controversial.

**Objectives:**

This study aimed to evaluate the impact of WLL on pulmonary function—specifically diffusion capacity—in patients with pneumoconiosis presenting with a baseline DLCO of less than 80% of the predicted value.

**Methods:**

This single-center study analyzed a subgroup of 27 patients (14.7%) with a pre-operative DLCO <80% predicted, drawn from a larger cohort of 184 pneumoconiosis patients undergoing WLL. Pulmonary function tests (DLCO, FVC, FEV1, and FEV1/FVC ratio) and St. George’s Respiratory Questionnaire (SGRQ) scores were evaluated at baseline, 1 month, and 3 months post-WLL. Changes in parameters were assessed using paired *t*-tests.

**Results:**

The analyzed cohort (*n* = 27; 24 males; mean age 49.0 ± 7.0 years) demonstrated a statistically significant improvement in DLCO % predicted at 1 month post-WLL compared to baseline (70.8 ± 8.1% vs. 74.1 ± 10.8%, *p* = 0.031). At 3 months, the improvement in DLCO % predicted (73.6 ± 12.1%) was no longer statistically significant relative to baseline (*p* = 0.074). Furthermore, no significant temporal changes were observed in FVC % predicted, FEV1% predicted, the FEV1/FVC ratio, or SGRQ scores across the follow-up periods.

**Conclusion:**

WLL yields a transient improvement in pulmonary diffusion capacity at 1 month in pneumoconiosis patients with pre-existing diffusion dysfunction; however, this benefit is not sustained at 3 months. Furthermore, the procedure does not significantly alter ventilatory function or health-related quality of life within this timeframe.

## Introduction

Pneumoconiosis, a spectrum of occupational lung diseases caused by the chronic inhalation of mineral dust, typically results in progressive pulmonary fibrosis and restrictive ventilatory deficits (1). A critical complication of this disease progression is impaired gas exchange. This is evidenced by a reduced diffusing capacity of the lungs for carbon monoxide (DLCO), which is observed in approximately 19.2% of pneumoconiosis patients, including 7.4% of those without a smoking history (2). Notably, DLCO impairment serves as an independent predictor of mortality in this population, correlating strongly with hypoxemic burden and subsequent cardiovascular complications (2, 3). Consequently, any therapeutic intervention capable of preserving or enhancing pulmonary diffusion capacity could theoretically improve the long-term prognosis for these patients.

Although whole lung lavage (WLL) was first introduced by Mason in 1982 for the management of pneumoconiosis, its overall therapeutic efficacy remains a subject of ongoing debate (4, 5). Accumulating clinical evidence suggests that WLL can alleviate respiratory symptoms, clearance of intra-alveolar dust and inflammatory cells, and temporarily improve ventilatory parameters with acceptable safety and tolerability (6). Experimental models have demonstrated that WLL can successfully remove retained dust particles (6), a mechanism that theoretically should improve diffusion capacity. In clinical practice, however, reports regarding the functional pulmonary benefits of WLL are highly conflicting (4, 5). To address this knowledge gap, the present study investigates the specific impact of WLL on DLCO and standard ventilatory parameters in pneumoconiosis patients with established diffusion dysfunction.

## Methods

This study was conducted in accordance with the Declaration of Helsinki and approved the Ethics Committee of the First Affiliated Hospital of Army Medical University (Southwest Hospital), located in Shapingba District, Chongqing, China (Approval No. KY2014047).

A total of 184 patients with pneumoconiosis undergoing whole lung lavage (WLL) were initially evaluated. Pulmonary function tests and St. George’s Respiratory Questionnaire (SGRQ) assessments were conducted at baseline (pre-WLL), as well as at 1-month and 3-month follow-ups. From this cohort, a subgroup of 27 patients (14.67%) was selected based on a pre-WLL diffusing capacity of the lungs for carbon monoxide (DLCO) of less than 80% of the predicted value. Statistical analyses were performed using SPSS software, employing paired *t*-tests to evaluate differences between time points.

## Results

Baseline characteristics of the 27 included patients are detailed in Table 1. The cohort comprised 24 males, with a mean age of 49.0 years and a mean body mass index (BMI) of 24.82 kg/m^2^. The mean duration of occupational dust exposure was 16.8 ± 8.8 years. Disease severity was classified as stage I (*n* = 9), stage II (*n* = 15), and stage III (*n* = 3).

**Table 1:** Demographic data of cases.

Characteristics	Value
Male (*n*)	24/27
Age (years)	49.33 ± 6.98
BMI (kg/m^2^)	24.82 ± 2.08
Dust exposure time (years):	16.8 ± 8.8
Stage of pneumoconiosis (*n*)	
I	9
II	15
III	3

**Figure 1:**
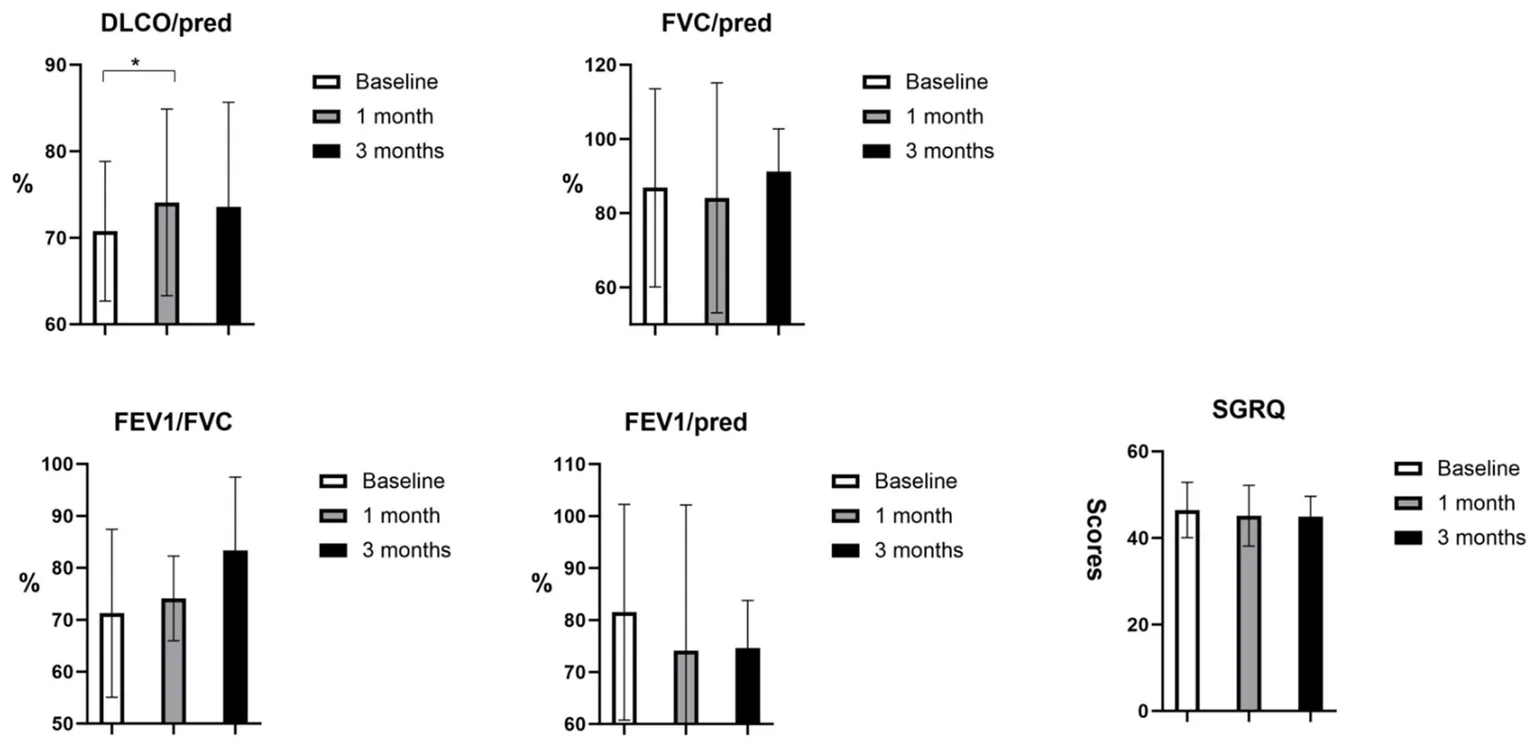
Results of lung function and St. George’s respiratory questionnaire scores before and after WLL. ^*^p < 0.05, SGRQ, St. George’s respiratory questionnaire scores. Diffusing capacity for carbon monoxide (DLCO), FVC (forced vital capacity), FEV1 (forced expiratory volume in 1 s), and St. George’s respiratory questionnaire (SGRQ).

Changes in pulmonary function and SGRQ scores are presented in Table 2, Figure 1. Mean DLCO % predicted (DLCO/pred) was 70.75 ± 8.07% at baseline, significantly improving to 74.09 ± 10.78% at 1-month post-WLL (*p* < 0.05). At 3 months post-WLL, DLCO/pred was 73.58 ± 12.08%; while higher than baseline, this difference did not reach statistical significance (*p* = 0.074).

**Table 2:** Results of lung function and St. George’s respiratory questionnaire scores before and after WLL (^*^p < 0.05).

TimeParameter	Baseline	1 month post-WLL	3 months post-WLL	*p* value baseline vs. 1 month post-WLL	*p* value baseline vs. 3 month post-WLL
DLCO/pred	70.75 ± 8.07	74.09 ± 10.78	73.58 ± 12.08	**0.031** ^*^	0.074
SGRQ scores	46.43 ± 6.39	45.13 ± 7.02	44.91 ± 4.67	0.232	0.191
FVC/pred	86.82 ± 26.71	84.12 ± 31.01	91.27 ± 11.45	0.655	0.323
FEV1/pred	81.49 ± 20.74	74.06 ± 28.06	74.57 ± 9.20	0.126	0.554
FEV1/FVC	71.23 ± 16.18	74.11 ± 8.16	83.33 ± 14.16	0.376	0.328

Ventilatory parameters showed no significant changes across the three time points (baseline, 1 month, 3 months, respectively): FVC/pred was 86.82 ± 26.71%, 84.12 ± 31.01%, and 91.27 ± 11.45%; FEV1/pred was 81.49 ± 20.74%, 74.06 ± 28.06%, and 74.57 ± 9.20%; and the FEV1/FVC ratio was 71.23 ± 16.18%, 74.11 ± 8.16%, and 83.33 ± 14.16%. Similarly, SGRQ scores were 46.43 ± 6.39 pre-WLL, 45.13 ± 7.02 at 1 month, and 44.91 ± 4.67 at 3 months, exhibiting a slight but non-significant downward trend.

## Discussion

Pneumoconiosis is an occupational lung disease caused by the inhalation of fine particulate matter (<5 micrometers), predominantly affecting workers in mining, construction, and manufacturing. The underlying pathophysiological mechanisms remain incompletely understood; however, even after the cessation of exposure, retained dust particles continue to damage lung tissue and drive disease progression (7, 8). Without timely intervention, patients risk severe complications, including intrathoracic infections, spontaneous pneumothorax, tuberculosis, chronic cor pulmonale, and respiratory failure (9–13).

Impaired pulmonary diffusion is highly prevalent in this population, even among non-smokers and those with normal ventilatory function (2). DLCO serves as a crucial indicator of gas exchange efficiency across the alveolar-capillary membrane (14), and acts as an independent prognostic factor, with reduced levels correlating to increased mortality risk (2, 3, 15, 16). DLCO <80% predicted defines diffusion dysfunction, which impairs O2 and CO2 transfer, potentially precipitating hypoxia, sleep-disordered breathing, and heart failure (17). Therefore, early detection and active intervention are vital.

Our findings demonstrate a significant, albeit transient, improvement in lung diffusion function at 1 month post-WLL among pneumoconiosis patients with baseline diffusion dysfunction. While DLCO continued to show numeric improvement at 3 months, it did not reach statistical significance (*p* = 0.074), which is likely attributable to an underpowered sample size. Unsurprisingly, WLL did not significantly alter ventilatory parameters (FVC, FEV1, FEV1/FVC) at either follow-up, as the lavage procedure clears alveolar debris but does not theoretically dilate fibrotic airways. Although SGRQ scores did not change significantly, a gradual downward trend was observed, suggesting a potential marginal benefit to quality of life that may require larger cohorts to validate.

## Conclusion

WLL yields a significant short-term improvement in pulmonary diffusion capacity (DLCO) at 1 month in pneumoconiosis patients with pre-existing diffusion dysfunction. Although a trend of improved DLCO persisted at 3 months, it was not statistically significant. WLL did not alter ventilatory function or significantly improve SGRQ scores; however, it remains a promising therapeutic modality that may positively influence the respiratory prognosis of these patients.

### Limitations

While this study is the first to document short-term DLCO improvements in this specific subgroup following WLL, several limitations must be acknowledged. These include a small sample size, the lack of a non-WLL control group, and an inability to perform stratified analyses based on disease stage or exposure duration. Furthermore, the 3-month observation period precludes conclusions regarding long-term efficacy (0.5–2 years). Future multi-center, randomized controlled trials are necessary to substantiate these findings.

## Data Availability

The original contributions presented in the study are included in the article/supplementary material, further inquiries can be directed to the corresponding authors.
